# Complete mitochondrial genome of a bat-eared fox (*Otocyon megalotis*), along with phylogenetic considerations

**DOI:** 10.1080/23802359.2017.1331325

**Published:** 2017-05-24

**Authors:** Michael Westbury, Fredrik Dalerum, Karin Norén, Michael Hofreiter

**Affiliations:** a Department of Mathematics and Natural Sciences, Evolutionary Adaptive Genomics, Institute for Biochemistry and Biology, University of Potsdam, Potsdam, Germany;; b Research Unit of Biodiversity (UO-CSIC-PA), Mieres Campus, University of Oviedo, Asturias, Spain;; c Department of Zoology, Stockholm University, Stockholm, Sweden;; d Department of Zoology, Mammal Research Institute, University of Pretoria, Pretoria, South Africa

**Keywords:** Phylogenetics, mitochondria, iterative mapping, Canidae

## Abstract

The bat-eared fox, *Otocyon megalotis,* is the only member of its genus and is thought to occupy a basal position within the dog family. These factors can lead to challenges in complete mitochondrial reconstructions and accurate phylogenetic positioning. Here, we present the first complete mitochondrial genome of the bat-eared fox recovered using shotgun sequencing and iterative mapping to three distantly related species. Phylogenetic analyses placed the bat-eared fox basal in the Canidae family within the clade including true foxes (*Vulpes*) and the raccoon dog (*Nyctereutes*) with high support values. This position is in good agreement with previously published results based on short fragments of mitochondrial and nuclear genes, therefore adding more support to the basal positioning of the bat-eared fox within Canidae.

The bat-eared fox (*Otocyon megalotis*) is a small member of the Canidae family and the only species of the genus Otocyon. It occurs in two allopatric populations across Africa (Clark 2005) and is considered a basal canid species (Sillero-Zubiri & Macdonald 2004). Studies using short mitochondrial and nuclear genes support a basal placement of the bat-eared fox within Canidae, as sister group to the clade including true foxes (*Vulpes)* and the Raccoon dog (*Nyctereutes procyonoides*) (Lindblad-Toh et al. 2005). However, despite previous genetics studies, the complete mitochondrial genome of the bat-eared fox has so far not been published.

Our bat-eared fox sample was captured on Benfontein game farm outside of Kimberley, central South Africa (28°99′ S, 24°81′ E, e.g. le Roux et al. (2014)) under permits from the animal care and use committee of the University of Pretoria (EC031-07) and from the provincial government in the Northern Cape (FAUNA 846/2009, FAUNA 847/2009).

DNA was extracted using a Zymo genomic DNA clean and concentrator extraction kit, built into Nextera Illumina sequencing libraries and sequenced on an Illumina Nextseq 500 (University of Potsdam, Germany). We trimmed raw reads using Cutadapt v1.4 (Martin 2011), merged overlapping fragments using FLASH v1.2.10 (Magoč & Salzberg 2011), and removed duplicate reads using Prinseq (Schmieder & Edwards 2011).

We undertook iterative mapping using MITObim v1.8 (Hahn et al. 2013) with default parameters apart from the mismatch value, which was 3%. Three independent runs were performed using different bait sequences from three species; *Canis lupus* (Genbank accession KC461238.1), *Vulpes vulpes* (Genbank accession JN711443.1), and *Urocyon littoralis* (Genbank accession KP128962.1). Consensus sequences were called using a minimum read coverage of 10× and a 75% base call threshold in Geneious v9.0.5 (Kearse et al. 2012). Automatic annotation was performed using MITOS (Bernt et al. 2013).

We aligned our sequence with representatives of Carnivora using Mafftv7.271 (Katoh & Standley 2013). We constructed a phylogenetic tree using BEAST version 1.8.4 (Drummond et al. 2012) on the Cipres server (Miller et al. 2010) specifying GTR I + G as the substitution model, as determined using Jmodeltest v2.1.7 (Posada 2008) and a Yule speciation model (Yule 1925; Gernhard 2008).

Our bat-eared fox mitochondrial sequence (Genbank accession KY776502) contains 16,431 bp. The genomes produced were identical regardless of starting bait reference. Our bat-eared fox mitochondrial assembly contained all 13 protein-coding genes with expected open reading frames, 22 transfer RNA genes, and 2 ribosomal RNA genes found within a typical vertebrate mitochondrial genome.

Phylogenetic analyses found further support for a basal placement of the bat-eared fox within the Canidae. *Otocyon* was placed with high confidence (posterior probabilities > 0.99) as sister to the clade containing both the raccoon dog (*Nyctereutes*) and true foxes (*Vulpes*) (Figure 1). This result is consistent with previous conclusions based on short regions of mitochondrial and nuclear genes (Lindblad-Toh et al. 2005).

**Figure 1.**
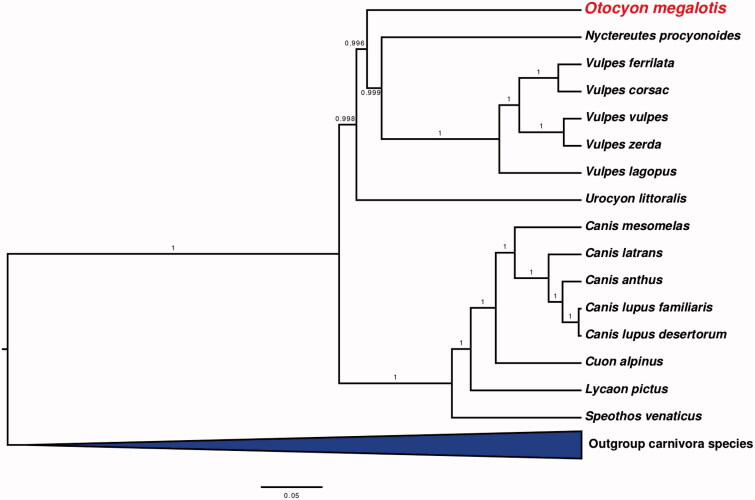
Bayesian tree showing the phylogenetic positioning of *Otocyon megalotis* within the Canidae and other clades within Carnivora. Numbers on branches represent posterior probabilities.
